# Personality Traits and Vitamin D3 Supplementation Affect Mood State 12 h Before 100 km Ultramarathon Run

**DOI:** 10.3389/fpsyg.2018.00980

**Published:** 2018-06-29

**Authors:** Daniel Krokosz, Mariusz Lipowski, Piotr Aschenbrenner, Wojciech Ratkowski

**Affiliations:** ^1^Department of Health Promotion, Faculty of Tourism and Recreation, Gdansk University of Physical Education and Sport, Gdańsk, Poland; ^2^Department of Natural Sciences, Faculty of Physical Education, Gdansk University of Physical Education and Sport, Gdańsk, Poland; ^3^Department of Management Tourism and Recreation, Faculty of Tourism and Recreation, Gdansk University of Physical Education and Sport, Gdańsk, Poland

**Keywords:** extreme-endurance sports, sport psychology, goals of sport, psychology of running, vitamin D3

## Abstract

**Background:** Participation in extreme endurance sports is becoming an increasingly popular activity, and thus more and more people are getting involved in it. Taking part in a 100 km run is associated with great physiological and psychological stress, which can affect one’s mood state. Thus, the goal of this study was to determine if personality, experience, and motives for participation are related to a runner’s mood and its changes as well as to investigate whether vitamin D3 supplementation influences mood 12 h before and 12 h after the run.

**Method:** The study group consisted of 20 experienced marathon and ultramarathon runners taking part in a 100 km track run. All participants were males aged between 31 and 50 (*M* = 40.75, *SD* = 7.15). The group was divided in two equal subgroups: the placebo group and the group supplemented with vitamin D3. Personality traits were assessed using the Polish version of Eysenck’s EPQ-R 106 and mood states were measured twice (12 h before and after the run) using the Polish version of the UMACL by Mathews, Chamberlain, and Jones. Motives for participation in ultramarathons were measured with the IPAO by Lipowski and Zaleski.

**Results:** Levels of vitamin D3 correlated very strongly with energetic arousal (EA) (*r_s_* = 0.80; *p* < 0.05) and strongly hedonic tone (HT) (*r_s_* = 0.74; *p* < 0.05) 12 h before the run. There were no significant correlations between levels of vitamin D3 and mood states after the run. Moreover, extraversion correlated moderately with tense arousal (TA) (*r_s_* = -0.48; *p* < 0.05) and EA (*r_s_* = 0.47; *p* < 0.05) while neuroticism correlated moderately with TA (*r_s_* = 0.53; *p* < 0.05) and HT (*r_s_* = -0.57; *p* < 0.05).

**Conclusion:** Both personality and vitamin D3 supplementation are related to runners’ pre-run mood. These effects are nullified when it comes to post-run mood states.

## Introduction

Until recently, extreme endurance sports were niche disciplines practiced only by a very small group of enthusiasts. They have grown in popularity in recent years, with more and more people engaging in this type of physical activity (Hoffman et al., 2007; Holt et al., 2014). This has been encouraged by an increasing number of running and triathlon events being organized – in 2017 over 20 races with distances of over 50k were organized in Poland (Polish Running Events, n.d.) and 306 such events were organized in the United States (Ultramarathon Calendar, n.d.). One particular type of endurance sport is ultramarathon (UM) running, which involves distances longer than 42.2 km (Knechtle, 2012; Ferrer et al., 2015). Such extended physical effort is an extremely strenuous activity, both physiologically (Millet et al., 2002; Knechtle, 2017; Zakovska et al., 2017; Knechtle and Nikolaidis, 2018) and psychologically (Rauch et al., 1988; Tharion et al., 1988; Nicolas et al., 2011). Research suggests that during a UM run participants are exposed to risks of overexertion, dehydration, gastrointestinal tract problems, and the associated psychological consequences (Bowen et al., 2006; Holt et al., 2014; Chlíbková et al., 2018). Whether taking part in this type of activity is natural for humans or whether it goes beyond a person’s normal biological and psychological constitution has been repeatedly questioned (Bramble and Lieberman, 2004; Pearson, 2006; Holt et al., 2014). These considerations make UMs a subject of interest for researchers in many areas of science, including psychology. Psychological research in this area usually concentrates on describing the motivation of participants (Hashimoto et al., 2006; Shipway and Holloway, 2010), mood of participants (Rauch et al., 1988; Tharion et al., 1988; Johnson et al., 2016), specific cognitive strategies used in order to finish a race (Acevedo et al., 1992), the phenomenological aspects of runners’ experiences during a race (Allen-Collinson and Hockey, 2011; Simpson et al., 2014) and their personality traits (Hughes et al., 2003; Martinez and Scott, 2016). The presented study aims to investigate which factors are related to a runner’s mood and its changes.

### Personality Traits of UM Runners

There is no doubt that UM running is an extreme sport in terms of both physical and psychological requirements, however, it is rarely described as a high-risk sport. An analysis of deaths during marathons shows that the probability of such a thing happening is very low – i.e., 0.8 for every 100,000 runners (Redelmeier and Greenwald, 2007; Mathews et al., 2012), although the plentiful media interest in such cases makes them seem more common. Research has repeatedly shown a specific personality profile for people engaging in extreme sports (Cazenave et al., 2007; Gomà-i-Freixanet et al., 2012; Krokosz, 2017), yet there is no such specific profile for people engaging in UM. Few temperament and personality studies have shown UM participants to have higher than normal results on the extraversion and openness to experience scales as well as the experience seeking scale (Hughes et al., 2003) This suggests that they are usually individuals who are interested in the world and open to new information. Importantly, no differences were noted between UM participants and the norm group on disinhibition as well as thrill and sensation seeking scales, which may suggest that ultramarathon runners do not seek stimulation through the use of psychoactive substances nor they seem eager to take part in typical extreme sports disciplines (Hughes et al., 2003). Moreover, Jack and Ronan (1998) showed that marathon runners score significantly lower an all subscales of the Sensation Seeking Scale V than do people who engage in extreme sports such as paragliding, parachuting, car racing, and mountain climbing. Potgieter and Bisschoff (1990) found that marathon runners assess their sport as less risky than rugby and scored lower on the thrill and adventure seeking scale than did rugby players. It should be noted, however, that marathons differ from UMs in terms of levels of strain and risk, and the question of how UM runners compare to individuals who engage in extreme sports remains open. In this context, it is particularly interesting to study what kind of goals motivate individuals who voluntarily undertake such extreme physical exertion.

### Motives for Participation in UMs

Research show that the main motives for taking part in UMs are no different from those most commonly reported for other forms of physical activity (Kilpatrick et al., 2005; CBOS, 2013; Lipowski and Zaleski, 2015; Lipowski and Ussorowska, 2018) and they are mainly related to a sense of personal achievement, taking care of one’s health, overcoming difficulties, as well as contact with nature (Doppelmayr and Molkenthin, 2004; Ferrer et al., 2015). Hashimoto et al. (2006) found out that apart from the motives mentioned above, meeting people and socializing with other runners were important motives for participation in UMs. Interestingly, UM runners often asses competition goals as less important than do other athletes, including marathon runners (Krouse et al., 2011; Kruger and Saayman, 2013). Extreme endurance athletes can be also motivated by achieving a positive emotional state called *runner’s high* which is related to flow experience (Weinberg, 1999). Factors concerning the emotions and moods of the runners can play an important role during the preparations and the participation in 100 km run.

### Mood States

The results of studies on mood state before and after a UM run are not surprising – after the run most runners have decreased results associated with vigor and mental stress, while indices of tiredness and exhaustion are increased (Rauch et al., 1988; Tharion et al., 1988). It is argued whether mood states of athletes can influence their performance to a significant extent (Lane, 2016). After conducting a meta-analysis, Rowley et al. (1995) pointed out that the mood state measured by Profile of Mood State Scale (McNair et al., 1971) is a questionable predictor, accounting for only 1% of variance in performance, leaving 99% of variance unexplained. On the other hand, Terry (1995) argues that mood has a subtle influence on performance and that this fact has to be taken to account when designing a study. Lane (2016) emphasizes that it is unquestionable that mood influences the thoughts and behavior of an athlete and thus can have a strong impact on their performance. Lane and Terry (2000) proposed a model suggesting that depressed mood is a moderating factor in the relationship between anger and tension with performance. While anger and tension can benefit performance when depression is absent, both are linked with poor performance when an athlete is experiencing depressed mood.

### Vitamin D3 Supplementation

An important aspect of research on taking part in UMs is the area concerned with the influence of supplementation on the physical condition of runners (Knechtle et al., 2012), however, the psychological consequences of the supplements are rarely considered. Studies and meta-analyses from fields not concerned with sports suggest that vitamin D3 has a positive influence on mood in humans and can reduce symptoms of depression, especially in the autumn–winter period when levels of sunlight (crucial for production of the vitamin in humans) are low (Lansdowne and Provost, 1998; Gowda et al., 2015; Sanaei et al., 2016). Research also shows a relationship between D3 levels and levels of perceived stress (Nowak et al., 2016). Taking into account the fact that in Central Europe, populations are on average deficient in vitamin D3, i.e., their serum levels are below 30 ng/ml (Pludowski et al., 2014), one may suppose that the consequences of deficiencies of the vitamin during the Autumn period may aggravate the negative consequences of running.

### The Present Study

Although personality traits, motives, and mood states have been investigated many times before (Roebuck et al., 2018), there is still a limited understanding of how they relate to each other in UM runners. Taking part in a UM is an extremely exhausting activity which can lead to both psychological and physiological stress and can have a very strong impact on the runners’ moods. The mood state itself can play a crucial role in athletes’ performance (Lane and Terry, 2000) and their well-being (Cummins, 2010). There is strong evidence that mood state can be related to personality traits such as extraversion and neuroticism (Matthews et al., 1990) but it is still unknown whether these traits can be considered risk factors or protective factors against pre-run and post-run negative mood states. Some motives for participation in UMs can also be related to mood states in runners. It is possible that runners who find socializing an important motive for participation in UMs can cope better with stressful situations by seeking social support. Since mood is significantly different before and after the run (Rauch et al., 1988; Tharion et al., 1988) and can be affected by different factors (e.g., neuroticism might be related to pre-run fear of injury but might not affect post-run mood state), it was important to measure the mood states of the runners 12 h before and 12 h after the 100 km run. As mentioned before, vitamin D3 is proven to have a positive influence on mood states and perceived stress (Nowak et al., 2016), but further investigation is needed to find out if the positive effect of supplementation can nullify negative pre-run and post-run mood states in ultramarathoners. Thus, the goal of the study was to determine the factors (personality traits, goals of participation, experience) related to a runner’s mood and its changes and to investigate if mood is influenced by vitamin D3 supplementation. Based on the aforementioned facts four hypotheses were made:

Hypothesis 1: in runners, extraversion will be associated with high scores on energetic dimensions of mood, and neuroticism will be associated with high levels of stress-related arousal.
Hypothesis 2: runners who asses goals concerning social interaction as important will have low levels of stress-related arousal before the run.
Hypothesis 3: more experienced runners will feel lower levels of stress-related arousal before the run.
Hypothesis 4: vitamin D3 supplementation will positively affect all dimensions of mood before and after the run.

## Materials and Methods

### Methods

The Polish adaptation (Jaworowska, 2011) of the Eysenck Personality Questionnaire EPQ-R (Eysenck et al., 1985) was used to assess the personality features of individuals taking part in the UM. This tool was developed based on Eysenck’s Personality Theory. The Polish adaptation is composed of the following scales: (1) extraversion, (2) neuroticism, (3) psychoticism, and (4) the lie scale; and two additional scales: (5) proneness to addiction, and (6) proneness to criminality. All of the above scales had Cronbach’s α reliability coefficient above 0.75. EPQ-R was chosen for this study because the measured scales are related to the biological functioning of humans. Moreover, the two most important scales for this research (extraversion and neuroticism) can be interpreted in terms of the functioning of the nervous system (central and autonomic), can be related to mood states, and are consistent with the interdisciplinary framework of this study.

The mood of the participants was assessed using the UWIST Mood Adjective Check List (UMACL) (Matthews et al., 1990) with Polish adaptation by Goryńska (2005). In this test, runners assessed the degree to which their present mood was described by each of the 29 listed adjectives on a scale from 1 to 4. The final score is represented by three dimensions: energetic arousal (EA), tense arousal (TA), and hedonic tone (HT). High levels of EA correspond to feelings such as being restful, energetic, and vigorous, while high scores of TA correspond to being stressed, anxious, or tense. HT is associated with being cheerful, satisfied, and happy (high scores), or concerned, depressed, and sad (low scores; Goryńska, 2005). Before completing the test, the athletes were asked about any potential situations unrelated to training that might affect their mood, but no such factors were declared.

The Inventory of Physical Activity Objectives (IPAO) by Lipowski and Zaleski (2015) was used in order to determine level of involvement in ultramarathon running. The respondent answers questions regarding their involvement in competitive sports (both present and previous) as well as the forms and intensity of their physical activity. The IPAO includes 12 objectives which are accompanied by a Likert scale (1–5), and the respondent is asked to assess the importance of the listed goals of participation, where 1 stands for completely unimportant and 5 for very important. In addition to measuring the attitude of the subjects toward particular goals, the individual scores on the Likert scales are summed up. The final score indicates the importance of the heterogeneity of the objectives one sets. The Cronbach’s α reliability coefficient for this version was 0.78.

### Participants

The study group consisted of 20 experienced marathon and ultramarathon runners taking part in a 100 km track run. All participants were male, aged between 31 and 50 (*M* = 40.75, *SD* = 7.15). They came from all over Poland and took part in the study at the location of the race. The study design, despite the organizational difficulties it presented, allowed all measurements to be controlled and the required tests to be efficiently conducted. Three participants did not finish the race, of whom two did not take part in the second measurement of mood. The participants’ experience in marathon and ultramarathon running ranged from 3 to 36 years (*M* = 11.05, *SD =* 10.60) and they had completed between 1 and 539 (!) marathons (*M* = 43.63) and between 1 and 35 (*M* = 11.95) ultramarathons prior to the study. It is worth noting that the individual who had only run one marathon had also finished five ultramarathons, and the person who had finished only one ultramarathon is an experienced marathon runner who had finished six marathons in the previous 3 years. Participants gave written consent to take part in the study and the study procedure was approved by the Local Ethical Board Committee (NKBBN/448/2016).

### Procedure

The study group was randomly divided in two equal subgroups: the placebo group and the experimental group (supplemented with vitamin D3). The study used a double-blind procedure. The groups were homogenous in terms of each of the investigated variables – there were no differences in vitamin D3 levels, age, experience, or personality features. Participants from the experimental group took 10000 IU of vitamin D3 (Vigantol^®^) daily for 2 weeks preceding the race, while individuals from the placebo group took sunflower oil. Supplementation was successful: the experimental group had significantly higher serum levels of D3 (*M* = 28.18 ng/ml; *SD* = 5.88 ng/ml) than did the placebo group (*M* = 20.41 ng/ml, *SD* = 4.99 ng/ml; *Z* = 2.36, *p* = 0.02). Levels of vitamin D3 were measured 12 h before the 100 km run. The psychometric tests were done at the same time. The race took place on a 400 m racetrack in a sports stadium. The race consisted of 250 laps in one direction; it started at 7.00 AM and the last participants finished around 9:00 PM. On the day of the race, the temperature ranged between 1 and 4°C, and atmospheric pressure ranged between 1000 and 1009 hPa. Participants had their blood taken every 25k of run in order to monitor the physiological strain. It is worth stressing here the extraordinary character of the effort that each participant put into taking part in the experiment. As well as the obvious effort associated with the UM running, the psychological difficulty was increased by the fact that the run was very monotonous (around the stadium) and was not associated with additional gains (monetary rewards or media attention). The participants were asked about their preferred drinks, meals, and energy bars, which were provided by the organizer and distributed during the race. The second mood measurement was taken the next day, 12 h after the participants finished the race. Mean finish time was *M* = 728.73 min (minimum = 589.62 min, maximum = 811.92 min, *SD* = 68.72 min). The times of mood measurement (12 h before and 12 h after) were selected such that they would cause the least nuisance to the runners and their preparation routine for the training, as well as to avoid interfering with their sleep following the run.

### Statistical Analysis

Statistical analysis was conducted using Statistica 12 software (repeated measures 2x2 ANOVA, *post hoc* analysis – Fisher’s least significant difference test, non-parametric Mann–Whitney *U*-test, and Spearman’s correlation).

## Results

### Personality Traits of Participants

In the first step, the personality profile of the participants was analyzed (**Table 1**). Importantly, there were no differences in personality features between the placebo and the vitamin D3 group, and so the results are presented together.

**Table 1:** Personality features of individuals who took part in the 100 km ultramarathon.

	*M* (*SD*)	*M* (*SD*) sten
Psychoticism	8.11 (3.63)	5.68 (1.83)
Extraversion	15.53 (5.09)	6.42 (2.48)
Neuroticism	7.63 (4.90)	4.84 (1.77)
Lie scale	10.16 (4.60)	5.05 (2.09)
Addiction	11.05 (2.84)	6.47 (0.70)
Criminality	10.21 (3.99)	5.26 (1.37)

**FIGURE 1:**
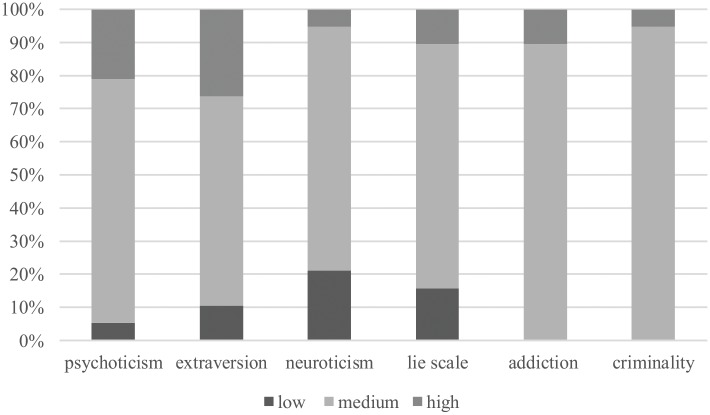
Levels of personality features in individuals taking part in the 100 km ultramarathon.

**Figure 1** illustrates the levels of personality features in the group of participants.

### Motives for Participation in UM Running

The most important motives for taking part in UMs were physical fitness, health, and pleasure from physical activity. The least important ones were boosting confidence and gaining appreciation from others. Assessments of the importance of each of the motivations for taking part in UMs are presented in **Table 2**.

**Table 2:** The importance of motivations for taking part in UM, as assessed by the participants.

Motivations for taking part in UM:	*M* (*SD*)
Physical fitness, being ‘in shape’	4.68 (0.58)
Health	4.63 (0.50)
Pleasure from physical activity	4.63 (0.50)
Well-being	4.47 (0.51)
Fulfilling the need for activity	4.16 (0.90)
Managing stress	4.05 (0.85)
Promoting PA by setting a behavior example	3.74 (1.15)
Escape from everyday life	3.68 (1.06)
Company of other people	3.58 (1.02)
Fit, shapely body	3.58 (1.02)
Being physically active and fit in order to be fashionable	3.05 (1.43)
Boosting confidence, gaining appreciation from others	3.05 (1.13)

### Mood States Before and After the 100 km Run

In the next step, the three dimensions of mood of the runners measured 12 h before and 12 h after the race in both groups were analyzed. A repeated measures 2x2 ANOVA [measurement × supplementation] was performed.

**FIGURE 2:**
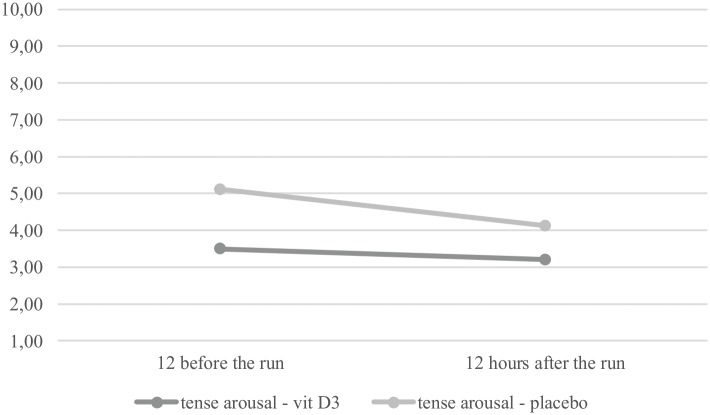
Levels of tense arousal (TA) before and after running in each group.

For TA it was found that the interaction effect was bordering significant, *F*(1,17) = 3.65, *p* = 0.07. The main effect of measurement (before or after the run) was significant, *F*(1,17) = 5.44, *p* = 0.03. Participants experienced higher TA 12 h before the run (*M* = 14.32, *SD* = 3.52) than after the run (*M* = 12.11, *SD* = 4.09), *post hoc: p* = 0.04. No group differences were observed for measurements before and after the run, though for the measurement before the run the difference was bordering on significant (*p* = 0.06). The relationship is presented in the context of sten norms in **Figure 2**. TA of the participants who supplemented with vitamin D3 was relatively low when compared with norms: before the run (*M =* 3.50, *SD* = 1.19) and after the run (*M =* 3.20, *SD* = 1.48). In the placebo group, this result was average at both measures: before the run (*M =* 5.11, *SD* = 1.54) and after the run (*M =* 4.12, *SD* = 1.88). These results indicate that the participants were not stressed by the 100 km run.

**FIGURE 3:**
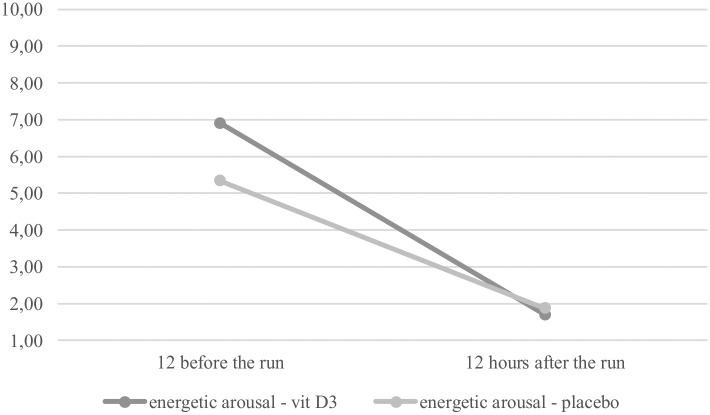
Changes in energetic arousal (EA) before and after the run in the two groups.

The results were similar with regards to EA – the interaction effect was insignificant, *F*(1,17) = 0.37, *p* = 0.55, but again there was a main effect of measurement time – before and after the run, *F*(1,17) = 83.37, *p* < 0.001. As might be expected, participants experienced significantly higher levels of EA 12 h before the run (*M* = 33.00, *SD* = 3.61) than 12 h after the run (*M* = 19.74, *SD* = 5.43), *post hoc: p* < 0.001 (**Figure 3**). There was also a significant between-group difference in EA before the run – individuals from the vitamin D3 group experienced significantly higher levels of EA (*M* = 34.60, *SD* = 3.13) than did individuals from the placebo group (*M* = 31.22, *SD* = 3.38; *Z* = 2.00, *p* = 0.04). When compared with norms, both groups experienced rather moderate EA before the run (supplemented group: *M =* 6.90, *SD* = 1.45; placebo group: *M =* 5.33, *SD* = 1.58). After the run, all of the participants were extremely fatigued and their results on the EA scale were close to minimal (supplemented group: *M =* 1.70, *SD* = 0.95; placebo group: *M =* 1.87, *SD* = 0.83).

For HT, no differences were observed for either time of measurement or between groups and the results of the participants oscillated around the upper border of the norms (*M* = 6.37, *SD* = 1.21 before the run; and *M* = 6.78, *SD* = 1.35 after the run) indicating that the runners were not depressed but rather slightly optimistic and happy.

### The Relationship Between Personality Traits, Goals of Participation, Experience, and Vitamin D3 Supplementation With Mood States Before and After the Run

In order to investigate the relationship between personality features and the mood of the runners, Spearman’s non-parametric analysis of correlations was performed. A clear, moderate relationship was found between two of the personality features (extraversion and neuroticism) and mood. Extraversion was positively correlated with EA 12 h before the start (*r_s_* = 0.47, *p* < 0.05) and negatively correlated with TA 12 h before the start (*r_s_* = -0.48, *p* < 0.05). An equally moderate relationship was observed in the case of neuroticism, which was correlated positively with TA before the run (*r_s_* = 0.53, *p* < 0.05) and negatively with HT before the run (*r_s_* = -0.57, *p* < 0.05). There were also some strong relations between vitamin D3 levels and mood before the run. These relationships concerned HT (*r_s_ =* 0.74, *p* < 0.05) and EA (*r_s_* = 0.80, *p* < 0.05).

No relationship was observed between the mood of the participants and their running experience.

However, moderate and strong relationships were observed between the importance of goals of participation in UMs and mood of participants before the run. The more important the motivation regarding the company of other people, the higher were the levels of EA (*r_s_* = 0.63, *p* < 0.05) and HT (*r_s_* = 0.56, *p* < 0.05). Unsurprisingly, the importance given to this motivation also correlated with extraversion (*r_s_* = 0.48, *p* < 0.05). A relationship was also observed between TA and the importance of two goals: managing stress (*r_s_* = 0.50, *p* < 0.05) and escape from everyday life (*r_s_* = 0.46, *p* < 0.05).

## Discussion

The presented study investigated what factors are related to the mood states of participants of a 100 km UM run. The results obtained indicate that personality traits, goals of participation in UM running, and vitamin D3 supplementation are related to the athletes’ mood states, but only before the run. Mood state after the run was not related to any of the factors examined.

In hypothesis 1 it was assumed that extraversion will be associated with high scores on EA and neuroticism will be associated with high levels of TA. The results obtained indicate that this hypothesis is only partially verified. The higher the levels of extraversion among the studied ultramarathon runners, the lower the stress they felt before the run and the higher their perceived energy and vigor before the run. This is consistent with the findings of Matthews et al. (1990), which indicate that extraversion is associated with increased sensitivity to reward signals and higher EA. This result may be also explained by the fact that extraverts are more likely to experience positive affect (Larsen and Ketelaar, 1991; Rusting and Larsen, 1997) and that they also seek social support, thus improving their mood (Lucas et al., 2008). The relationship between neuroticism and the tension perceived before the race as well as lowered levels of perceived happiness may also be explained by the nature of this personality feature, which is associated with the tendency to experience negative emotions (Rusting and Larsen, 1997) and increased sensitivity to punishment signals (Matthews et al., 1990). Importantly, although those relationships were relatively strong and are consistent with the previous research, they vanished completely 12 h after the race. It might be the case that the psychological nature of these relations is so subtle that physiological changes presented in meta-analysis by Knechtle and Nikolaidis (2018), such as: muscle damage, kidneys disfunction, digestive tract problems, increase of cortisol level, which took place during and after the race, dominated the moods of the runners. Acquired result can be also explained by the possible depletion of mental resources from the exertion of self-control during the 100 km run. Muraven and Baumeister (2000) suggest that long term tasks that require either coping with stress, regulating mood, or resisting temptation (e.g., to discontinue participation in the 100 km run) tend to weaken self-control strength after the task. This could have nullified the impact of psychological traits on the runners’ moods.

It was also hypothesized that runners who assess goals concerning social interaction as important will have low levels of stress-related arousal before the run (hypothesis 2). The results obtained indicate that this assumption was wrong. TA was not related to the importance of socialization goals, but EA and HT were correlated significantly with this motive. The more important were these goals, the happier and more vigorous were the runners. This finding is consistent with the work of Feeney and Collins (2015), who propose a new look at thriving through social relationships. Not only are these relationships associated with dealing with life’s adversities, but more importantly, they play a crucial role in exploration and growth. The results obtained may also be explained by the relationship of the importance of socializing goals with extraversion. Extroverted runners can enhance their mood by achieving social interaction goals.

The third hypothesis assumed that the more experienced the runners (in terms of years of running or number of completed races), the lower the levels of stress-related arousal they will feel before the run. It may seem surprising that this hypothesis was also proven to be wrong. It could be that the reasons for feeling tension differ between experienced and less experienced runners. Experienced runners may be anxious because they can remember the negative outcomes of such extreme physical effort, and those with less experience may be stressed by the fact that they cannot foresee how their organism will react to the circumstances of the race. On the other hand, all of the participants were experienced runners and it is possible that a ceiling effect of experience occurred. The relatively low results in TA obtained by all participants before the run might confirm this interpretation.

The final hypothesis assumed that vitamin D3 supplementation will positively affect all dimensions of mood before and after the run. Once again, this hypothesis was only partially confirmed by the results. Interestingly, the relationship between D3 levels and mood was significant only for the pre-race mood of the participants – the higher the serum D3 levels, the more energetic and vigorous they felt (EA) and the more content and happy they felt (HT). This result is in line with previous research, which indicated that vitamin D3 has a positive influence on mood and an ability to decrease the symptoms of seasonal depression (Berk et al., 2007). Again, no relationships were observed in the context of TA before the run. As mentioned before, both groups of participants were not distressed before the run so the possible positive effect of vitamin supplementation could have been insignificant. D3 levels had no relationship with the subjective perception of fatigue 12 h after the run – it seems that the opposite of a ceiling effect occurred: the exhaustion was so severe that nothing alleviated its levels (in comparison to the norms, the results were, on average, around the 2nd sten). It is worth noting here that the race discussed in this study happened in Autumn, in unfavorable weather conditions. Despite the fact that the participants spent a significant amount of time outdoors, their serum D3 levels were below the reference value, i.e., below 30 ng/ml (Płudowski et al., 2013). Unfortunately, the obtained correlational results were not confirmed by ANOVA comparisons of means. This could be explained by the main limitation of this study – the sample size; hence, inferences about the influence of vitamin D3 on runners’ moods should be confirmed in future studies.

As well as verifying the hypotheses, descriptive statistics revealed interesting observations. Results of studies concerning personality do not provide a clear profile characteristic to UM participants. A slightly higher level of extraversion is in line with the results of Hughes et al. (2003). In the current group of participants, there were as many as five people with a high result on that scale and only two with a low result. In line with Eysenck’s (1973) theory, high extraversion scores may indicate the higher need for stimulation usually associated with extreme sports (Gomà-i-Freixanet et al., 2012). Despite the fact that UM runs can put extreme strain on one’s body, their nature is entirely different from that of, say, B.A.S.E. jumping. In the case of UM running, the ‘extreme’ is associated with the ability to detach oneself from perceived pain and exhaustion (Simpson et al., 2014), rather than with concentrating on positive and exciting experiences, as is usually the case with the more ‘traditional’ extreme sports (Buckley, 2012). However, a certain type of journey within oneself in order to get to know one’s limits may be the common ground between these disciplines (Simpson et al., 2014), as it is also often mentioned in accounts of the experiences of extreme sportsmen (Brymer and Schweitzer, 2017).

The results regarding mood confirm previous findings (Rauch et al., 1988; Tharion et al., 1988) in terms of a clear reduction in levels of vigor and energeticness, as well as the psychological tension associated with the run. It is worth noting that TA before the race was not high in comparison to the norms – the results oscillated around the fifth sten. Moreover, mood observed 12 h before the race was not related to the finishing time achieved the following day in the 100 km race. It is difficult to talk about comparing the results with personal bests because of the particularities of this race: the need to stop every 25k for physiological assessments, the weather conditions, and the fact that the race took place on a stadium track. It is worth mentioning that the fact that the run was 250 laps of a track could also be a factor related to the runners’ moods. These circumstances could have lowered the mood of some runners, especially those who consider running in nature to be an important motive for participation in UMs.

The analysis of the motivations driving the runners is in line with the results of studies which have indicated that goals are related to health and well-being (Krouse et al., 2011). These motivations are no different from those listed by people who engage in disciplines less demanding than UM (Lipowski and Ussorowska, 2018). At first glance this result may be surprising, as the immediate effects of the competition don’t have beneficial impact on runners’ health, but as Knechtle and Nikolaidis (2018) point out, the overall effect caused by long-term endurance training is positive for athletes health.

It is worth noting that the current study has a significant limitation – i.e., the small number of participants. This made it impossible to perform more sophisticated statistical analyses or to extrapolate the results to the general population. However, it should be remembered that this is a small and hard-to-reach population, and conducting a study at a single time in a single place is likely to remain a challenge.

## Conclusion

It has been shown that attention must be paid to factors which may influence the mood of runners before taking part in physical activity that puts extreme strain on their organism. The beneficial effects of vitamin D3 supplementation on positive mood before the race have been confirmed, as were the relations of extraversion and neuroticism with pre-race mood. Paying attention to the emotional consequences of taking part in races may translate into improvement of runners’ well-being.

## Author Contributions

DK, ML, and WR contributed conception and design of the study. DK and PA organized the database and performed the statistical analysis. WR and DK organized funds for the study. DK wrote the first draft of the manuscript and wrote sections of the manuscript. All authors contributed to manuscript revision, read, and approved the submitted version.

## Conflict of Interest Statement

The authors declare that the research was conducted in the absence of any commercial or financial relationships that could be construed as a potential conflict of interest.
